# The Brain Endocannabinoid System is Differentially Regulated in Male and Female Patients with First-Episode Psychosis

**DOI:** 10.1093/schbul/sbag038

**Published:** 2026-05-05

**Authors:** Heikki Laurikainen, Reetta-Liina Armio, Lauri Tuominen, Mikko Nyman, Anna Kirjavainen, Johan Rajander, Merja Haaparanta-Solin, Olof Solin, Jarmo Hietala

**Affiliations:** Turku PET Centre, University of Turku and Turku University Hospital, 20521, Turku, Finland; Department of Psychiatry, University of Turku and Turku University Hospital, 20520, Turku, Finland; Turku PET Centre, University of Turku and Turku University Hospital, 20521, Turku, Finland; Department of Psychiatry, University of Turku and Turku University Hospital, 20520, Turku, Finland; Department of Psychiatry, University of Turku and Turku University Hospital, 20520, Turku, Finland; University of Ottawa Institute of Mental Health Research, K1Z 7K4, Ottawa, ON, Canada; Department of Psychiatry, Faculty of Medicine, University of Ottawa, K1Z 7K4, Ottawa, ON, Canada; Department of Radiology, Turku University Hospital & University of Turku, 20521, Turku, Finland; Turku PET Centre, University of Turku and Turku University Hospital, 20521, Turku, Finland; Accelerator Laboratory, Turku PET Centre, Åbo Akademi University, 20521, Turku, Finland; Turku PET Centre, University of Turku and Turku University Hospital, 20521, Turku, Finland; Turku PET Centre, University of Turku and Turku University Hospital, 20521, Turku, Finland; Turku PET Centre, University of Turku and Turku University Hospital, 20521, Turku, Finland; Department of Psychiatry, University of Turku and Turku University Hospital, 20520, Turku, Finland

**Keywords:** sex difference, early psychosis, CB1-receptor

## Abstract

**Background and Hypothesis:**

A sex difference in the clinical presentation of schizophrenia is well known. Males have on average an earlier symptom onset, worse functional capacity, and more negative symptoms. Studies on the neurobiological correlates of psychosis show that brain endocannabinoid system (ECS) is dysregulated in male patients with first-episode psychosis (FEP). We now evaluated whether the brain ECS is also altered in female patients with FEP.

**Study Design:**

In this cross-sectional case–control study, brain CB1R availability was measured in 39 participants, including groups of male and female patients with FEP, and healthy control participants (HC) of similar age and sex (*n* = 8-11/group). Brain CB1R availability was measured with the selective CB1R radiotracer [^18^F]FMPEP-*d*_2_ and positron emission tomography. Arterial input derived distribution volumes (V_T_) were extracted from regions of interest (ROI) representing the anterior cingulate cortex, hippocampus, thalamus, and putamen.

**Study Results:**

Within-subjects analyses showed a regionally differential effect of ROI*sex*group (ε = 0.77; *F*(2.31,80.85) = 4.31, *P* = .013). Simple effect analyses indicates that male FEP had significantly lower overall CB1R V_T_ when compared to male HC (*F*(1,17) = 15.64, p_FWER_ = 0.018), while female FEP V_T_ did not differ from female HC (*F*(1,18) = 0.12, p_FWER_ = 1). A regionally specific difference of V_T_ between males and females with FEP (*F*(3,48) = 3.43, *P* = .024) did not survive the correction for multiple comparisons (p_FWER_ = 0.14).

**Conclusions:**

The availability of brain CB1R is differentially altered in males and females with early psychosis. Sex-related neurobiological patterns including the ECS may offer new treatment strategies for alleviating the core symptoms of psychotic disorders in male and female patients.

## Introduction

Psychotic disorders form a group of common but severe psychiatric illnesses presenting with symptoms such as distortion of thought, and altered perceptions, emotions and language. The course of illness and treatment responses of psychotic disorders are individually variable, and sex differences of the epidemiology, and clinical presentation of psychotic disorders have also been described previously.^1^^,^^2^ Male patients with schizophrenia-spectrum psychoses have on average worse premorbid levels of functioning, a greater severity of negative symptoms, more cognitive dysfunction, lower responses to antipsychotic medication, and they also present with psychosis at an earlier age when compared to females.^1^^,^^3–5^ Functional outcome is also somewhat dependent on sex in patients with early psychosis.^1^ Characterizing the causes of these sex differences could shed light on the biological etiology of psychotic disorders.

Available pharmacological treatments targeting the dopaminergic system are effective in alleviating the dimension of positive symptoms, but negative symptoms remain resistant to treatment.^6^^,^^7^ Consistently, a dysregulation of midbrain dopaminergic projections is thought to underlie the formation of positive symptoms.^8^ This dysregulation is present in samples containing both male and female

patients, but with little evidence of any sex differences.^9^^,^^10^ Dopamine transporter function has been suggested to be one of the possible sex-specific factors affecting the impact of dopamine dysregulation in psychosis.^11^ Sex differences are also present in the endocannabinoid system (ECS), and consequently it presents as a putative explanation for the sex differences seen in psychotic disorders.^12–15^

The ECS is a ubiquitous signaling system involved in regulating the immunology, glial cells and neural functions in the brain, and also immune, metabolic and endocrine functions in the periphery.^16–22^ The availability of brain type 1 endocannabinoid receptor (CB1R) has been shown to be lower in male FEP patients compared to male HC irrespective of antipsychotic medication use.^14^^,^^15^ The increased in vivo binding of the selective CB1R radiotracer [^18^F]MK-9470, in a combined sample of male and female patients with chronic schizophrenia, further suggests that the ECS is dysregulated in both males and females.^23^ Elevated cerebrospinal fluid levels of the endocannabinoid anandamide in male and female psychosis patients also suggests an etiological role for the ECS in schizophrenia of both sexes.^24^ However, these prior studies have not accounted for the effects of sex, or the use of hormonal contraception, although these present as plausible confounders given that the ECS is regulated by gonadal hormones.^22^^,^^25^ Emerging evidence suggests that these factors could significantly affect measurements of ECS function.^26^^,^^27^ The ECS also contributes to the effects of mesolimbic dopamine signaling, which suggests an etiological contribution to the core symptomatology of psychoses, and several reports suggest that the ECS is indeed dysregulated in both first-episode and chronic psychotic disorders.^12–15^^,^^23^^,^^26^^,^^28–30^ Consistently, the ECS has been suggested to be a promising target for novel pharmacological treatment strategies of psychotic disorders. For example, the preventive effectiveness of cannabidiol in clinical high-risk for psychosis states is under study in a multi-site randomized trial.^31^ However, it is not currently known whether the ECS is similarly affected by psychosis in both sexes. Insight into the sources of variability in ECS function, such as due to sex, is important for understanding the etiology of psychosis, as well as for the design and interpretation of clinical trials.

We investigated whether a brain CB1R dysregulation is similarly present in male and female patients with first-episode psychosis (FEP), when compared with healthy control (HC) groups of similar age and sex. Based on previous clinical and preclinical studies indicating sex differences in ECS function,^32^ our primary hypothesis was that the changes in CB1R availability in vivo are more robust in males compared to females with FEP.

## Methods

In this study sex was defined as the sex assigned at birth. Sex was determined by interview and according to the Finnish national identification number. All participants were of Finnish ancestry. The study was approved by the ethics committee of Southwest Finland (ETMK 98/108/2013), and the Hospital Districts of Southwest Finland and Satakunta. Steps to ensure the confidential handling of data were approved by the ethics committee of Southwest Finland, and the Hospital Districts of Southwest Finland and Satakunta. The capacity for consent of patient participants was evaluated by the treating physician and first author. All participants gave their written informed consent prior to any study procedures. This study was conducted in accordance with the principles of the Declaration of Helsinki.

Healthy control participants between 18 and 40 years of age were recruited via the national population registry, a local newspaper advertisement, and an advertisement in local educational institutions between 2013 and 2022. The somatic status of all participants was confirmed by medical examination, blood and urine tests, electrocardiography, and MRI. The presence of psychiatric disorders was evaluated using the structured clinical interview for the diagnostic and statistical manual of mental disorders 4th ed. (DSM-IV) axis I disorders. Lifetime substance use was documented, and current use was controlled with a urine screen prior to the positron emission tomography (PET) scan for brain CB1-receptor availability. Exclusion criteria for the HCs were: (1) any lifetime Axis I-disorder, (2) lifetime general medical disorder or injury affecting the brain, (3) illicit substance use within 2 months prior to PET scanning, (4) pregnancy, and (5) use of any oral hormonal contraception. Altogether 11 male and 10 female HCs and were included in the statistical analyses. Results from the 11 male HCs have been previously reported^14^^,^^26^  Table 1.

**Table 1:** Demographics and Auxiliary Information.

Characteristics	Healthy volunteers (m;f)	Patients with FEP (m;f)	Test statistic FEP vs. HC (m; f)	df FEP vs. HC (m; f)	*P*-value FEP vs. HC (m; f)	Test statistic FEP male vs. female	df FEP male vs. female	*P*-value FEP male vs. female
*n*	11; 10	8; 10	NA; NA	NA; NA	NA; NA	NA	NA	NA
Age, y, mean (SD)	27.60 (5.84); 26.75 (8.95)	26.84 (3.86); 28.76 (5.41)	W = 46; W = 69	NA; NA	.90; .17	W = 51	NA	.36
Race/ethnicity, No. white/other	11/0; 10/0	8/0; 10/0	NA; NA	NA; NA	NA; NA	NA	NA	NA
Educational attainment, y, mean (SD)	15.73 (3.17); 15.45 (2.63)	13.29 (1.38); 15.85 (2.98)	W = 20; W = 54	NA; NA	.10; .79	W = 56.5	NA	**.04**
Frequency of alcohol use, No., none/<1 per mo/2-4 per mo/2-3 per wk/≥4 per wk	0/3/5/2/1; 0/4/5/1/0	1/5/1/1/0; 1/4/4/1/0	NA; NA	NA; NA	.24; 1.00	NA	NA	.81
Current tobacco use, yes/no	1/10; 1/9	4/4; 4/6	NA; NA	NA; NA	.11; .30	NA	NA	1
Lifetime cannabis use, No. using 0/1-5/6-10/11-50/>50 joints	3/5/2/1/0; 8/2/0/0/0	4/1/0/1/2; 8/1/0/1/0	NA; NA	NA; NA	.17; 1.00	NA	NA	.60
Diagnosis, No. with non-affective/affective psychosis	NA	5/3; 8/2	NA; NA	NA; NA	NA; NA	χ^2^ = 0.678	1	.41
Chlorpromazine equivalent dose, mean (SD), mg/day	NA	201.25 (193.79); 304.63 (247.44)	NA; NA	NA; NA	NA; NA	t = 0.966	16	.35
BPRS sum score, mean (SD)	NA	59.62 (16.79); 43.80 (12.11)	NA; NA	NA; NA	NA; NA	t = 2.24	12.37	**.04**
BPRS positive symptom sum (SD)	NA	7.88 (4.05); 4.7 (1.89)	NA; NA	NA; NA	NA; NA	W = 18	NA	.05
BPRS negative symptom sum (SD)	NA	19.38 (6.26); 14.70 (3.58)	NA; NA	NA; NA	NA; NA	t = 1.71	16	.11
Beck Depression Inventory (BDI) sum score, mean (SD)	1.45 (1.97); 1.00 (1.22)	14.57 (8.5); 17.25 (14.73)	W = 75.00; W = 65.50	NA; NA	**<.01**; **<.01**	t = 0.422	13	.68
Beck Anxiety Inventory (BAI) sum score, mean (SD)	1.82 (2.89); 2.00 (1.73)	15.00 (10.49); 17.11 (16.90)	W = 73.00; W = 73.00	NA; NA	**<.01**; **<.01**	W = 33	NA	.92
BMI, kg/m^2^, mean (SD)	25.25 (3.73); 24.46 (2.76)	28.25 (6.88); 23.32 (2.79)	W = 50.00; W = 34	NA; NA	.66; .24	W = 19	NA	.07
Injected dose, MBq, mean (SD)	199.64 (13.00); 203.60 (17.28)	203.25 (8.17); 202.9 (9.02)	t = 0.74; t = 0.11	16.75; 13.57	.47; .91	t = 0.086	15.7	.93

Values of *P* < .05 in bold. Abbreviations: FEP, first-episode psychosis; HC, healthy control; SD,; BPRS, Brief psychiatric rating scale.

First-episode psychosis patients between 18 and 40 years of age were recruited from psychiatric inpatient wards and outpatient clinics of Turku health services and the Hospital Districts of Southwest Finland and Satakunta. First-episode psychosis was defined as the presence of DSM-IV Axis I psychosis diagnosis with the onset of treatment for first episode of symptoms within 2 years of inclusion. Exclusion criteria for the FEP patients were: (1) current Axis I substance dependency disorder, (2) lifetime general medical disorder or injury affecting the brain, (3) illicit substance use within 2 months prior to PET scanning, (4) pregnancy, and (5) use of any oral hormonal contraception. Altogether 8 male and 10 female FEP patients were included in the statistical analyses. Results from 7 male FEP patients have been previously reported^14^  Table 1.

[^18^F]FMPEP-*d*_2_ was synthesized as described previously with slight modifications.^33^ The radiochemical purity was greater than 95% and the molar radioactivity greater than 500 GBq/μmol at the end of synthesis.

CB1R availability was indexed by the V_T_ of the selective radiotracer [^18^F]FMPEP-*d*_2_ and PET. Logan plot analysis was used to model V_T_.^34^ For 36 participants a T1-weighted MRI was acquired using the 3 Tesla Philips Ingenuity PET/MR scanner (Repetition time (TR) = 8.1 ms, Echo time (TE) = 3.7 ms, flip angle 7°, Field of view (FOV) = 256 × 256 × 176 mm^3^, voxel size 1 × 1 × 1 mm^3^). For 3 female FEP participants the T1-weighted MRI was acquired using the GE Signa PET/MR scanner (TR = 8.5 ms, TE = 3.7 ms, flip angle 10°, FOV = 256 × 256 × 200 mm^3^, voxel size 1 × 1 × 1 mm^3^). Cortical reconstruction and volumetric segmentation were done with the Freesurfer image analysis suite v. 6.0 (http://surfer.nmr.mgh.harvard.edu/). The rostral and caudal anterior cingulate cortex (ACC), hippocampus (HIPP), thalamus (THA), and putamen (PUT), as defined by the Desikan–Killiany atlas, were selected as regions of interest due to their putative involvement of the ECS within functionally interconnected circuits implicated in psychotic disorders, and to approximately replicate the regions analyzed in Borgan, Laurikainen et al.^14^^,^^35^^,^^36^ The synthesis of [^18^F]FMPEP-*d*_2_, MRI and PET procedures, analyses of radiometabolites, imaging and blood data preprocessing, and modeling of V_T_ were done as described previously in Laurikainen et al.^26^ Brief psychiatric rating scale (BPRS) positive symptom sub-scores were calculated by summing individual positive symptom severity ratings.^37^^,^^38^

The normality of regional V_T_ data was assessed using Shapiro–Wilk normality tests. Possible outliers were identified using Rosner’s test with 1 or 2 assumed outliers. Main effects and within-subject interactions were tested using repeated measures analysis of variance (rmANOVA). Greenhouse–Geisser sphericity corrections were used when applicable.

The following omnibus model was used to test the primary hypothesis:

A. All participants: V_T_ ~ ROI*sex*group + Error (ID/ROI).

The following secondary models were designed to examine the influence of specific simple effects [in brackets] underlying the interactions identified in Model A.:

B.1. Female participants: V_T_ ~ ROI*group + Error (ID/ROI), [group, ROI*group];

B.2. Male participants: V_T_ ~ ROI*group + Error (ID/ROI), [group, ROI*group];

B.3. First-episode psychosis patients: V_T_ ~ ROI*sex + Error (ID/ROI), [sex, ROI*sex].

Correction for multiple comparisons in test family B.1.–B.3., and in regional post-hoc tests, was done using Bonferroni family-wise error rate (FWER) correction. Corrected *P*-values are reported as p_FWER_.

All statistical testing was done using R version 4.3.1 (2023-06-16) “Beagle Scouts” and RStudio 2023.06.1+524 “Mountain Hydrangea” release for macOS.

## Results

### Basic Clinical Characteristics of the Study Samples

The study sample consisted of male (*n* = 8) and female (*n* = 10) patients with FEP, and male (*n* = 11) and female (*n* = 10) HCs matched by age and sex. There were no significant differences of age, current cannabis use, BMI or injected [^18^F]FMPEP-*d*_2_ dose between the males and females in either the HC or FEP groups, or between the females in the HC and FEP groups. Male FEP patients had a significantly higher total BPRS symptom sum score at the time of PET imaging when compared to female FEP patients. One male with FEP, and 2 females with FEP were not using antipsychotic medications at the time of the PET study. There were no differences in chlorpromazine equivalent defined daily doses in males and females with FEP.^39^

### CB1 Receptor Availability in FEP and HC Groups

**Figure 1:**
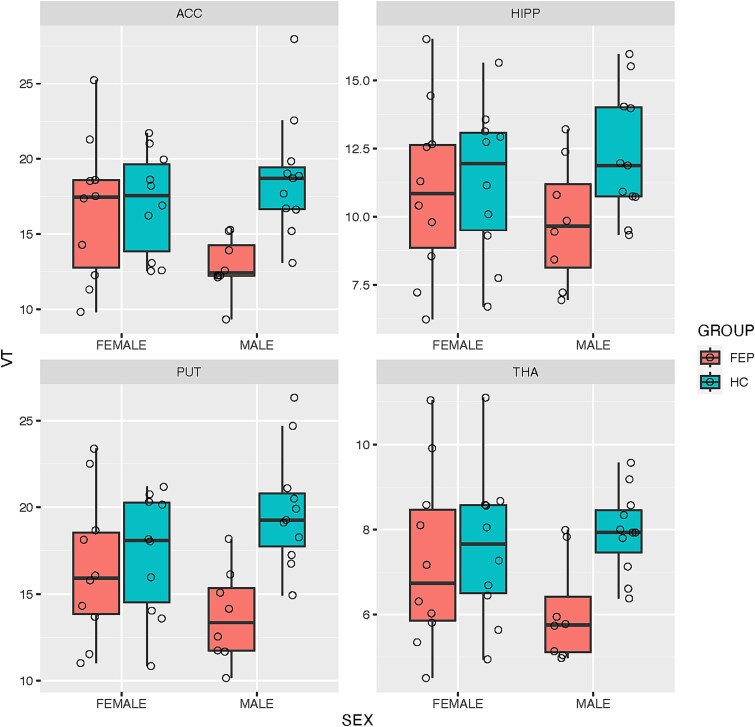
Regional differences of [18F]FMPEP-d2 VT in male and female FEP patients and HCs. Open circles represent values of individual observations (○). Bars indicate mean values. Whiskers indicate 95% confidence intervals. ACC, anterior cingulate cortex; FEP, first-episode psychosis; HC, healthy control; HIPP, hippocampus; PUT, putamen; THA, thalamus; VT, [18F]FMPEP-d2 distribution volume.

The rmANOVA model including all available participants revealed statistically significant interactions of ROI*sex*group (ε = 0.77; *F*(2.31,80.85) = 4.31, *P* = .013), and sex*group (*F*(1,35) = 4.30, *P* = .045). Covarying for the change in MRI scanner for 3 female FEP participants did not alter the results (*P* < .05). There were also statistically significant differences in [^18^F]FMPEP-*d*_2_ V_T_ between FEP and HC groups (*F*(1,35) = 6.87, *P* = .013), and between regions (*F*(3,105) = 344.21, *P* < .001). Cross-validation tests showed that the main group effect and ROI*sex*group interaction were not sensitive, and the sex*group interaction was sensitive to leaving out any one participant from the sample (*P* < .05) Figure 1.

Males with FEP had a significantly lower overall [^18^F]FMPEP-*d*_2_ V_T_ compared to the male HC (group; *F*(1,17) = 15.65, p_FWER_ = 0.018). This effect was significantly different between regions (ROI*group; *F*(3,51) = 10.11, p_FWER_ < 0.001). There were no differences of overall [^18^F]FMPEP-*d*_2_ V_T_ between female FEP and female HC (group; *F*(1,18) = 0.12, p_FWER_ = 1), and there was no regional interaction (ROI*group; ε = 0.691; *F*(2.07,37.31) = 0.10, p_FWER_ = 1). The rmANOVA with a subset of only FEP showed a regionally specific difference between males and females with FEP (ROI*sex; *F*(3,48) = 3.43, *P* = .024), but it did not survive correction for multiple comparisons (p_FWER_ = 0.14). Cross-validation tests also showed that this sex*ROI interaction was sensitive to leaving out any one participant from the sample (*P* > .05). Post-hoc estimated marginal means indicated a trend towards lower [^18^F]FMPEP-*d*_2_ V_T_ in males compared to females with FEP in the ACC (M_female_-M_male_ = 3.76, p_FWER_ = 0.072), but not in the PUT (M_female_-M_male_ = 2.80, p_FWER_ = 0.28), THA (M_female_-M_male_ = 1.22, p_FWER_ = 1) or HIPP (M_female_-M_male_ = 1.18, p_FWER_ = 1).

Histogram analysis and Shapiro–Wilk normality tests showed that the [^18^F]FMPEP-*d*_2_ V_T_ of ACC, HIPP, PUT, and THA were normally distributed with no outliers. There were no significant differences between group or sex in an rmANOVA model of unchanged tracer fractions (fraction ~ time*sex*group + Error (ID/ROI)). The area under curve of plasma [^18^F]FMPEP-*d*_2_ activity was significantly smaller in females when compared to males (*P* < .001), but not in FEP participants compared to HC (*P* = .194). The volume of the ACC ROI was on average 1442 mm^3^ smaller in FEP compared to HC (*P* = .006), and the volumes of ACC and THA ROIs were respectively on average 2442 mm^3^ (*P* < .001) and 1525 mm^3^ (*P* = .003) smaller in females when compared to males. There were no significant main effects of sex, group status, or sex*group interaction in an ANOVA model for in-scanner cumulative movement (*P* > .05).

### BPRS Symptoms in the FEP Group

Both male and female FEP patients had similarly low BPRS positive symptom severity scores (BPRS_positive_). Females has significantly lower total BPRS scores. Female FEP patients had higher mean scores in self-report scales for depression and anxiety symptoms when compared the mean scores of male FEP patients, but the differences were not significant. The female and male FEP groups had similar ratios of non-affective and affective psychotic disorders. Exploratory analysis results of the effects of BPRS_positive_ and BPRS_total_ on [^18^F]FMPEP-*d*_2_ V_T_ can be found in the supplement Table 1.

## Discussion

Our results indicate that the ECS is differentially involved in the etiology of psychotic disorders of males and females. Our findings extend the previous literature by showing that a robust ECS dysregulation is present in males, a significant difference is not present between female FEP and HC participants, and that the availability of CB1R is regionally different in males and females with FEP.^14^^,^^15^^,^^23^

Patients with schizophrenia have been suggested to have an altered balance of excitatory and inhibitory neurotransmission in the frontal cortex, the striatum, and the thalamus.^40^ In their article, Howes, Bukala, Beck^40^ review evidence supporting a hypothesis according to which decreased inhibitory signaling in the cortex disinhibits cortical projections to midbrain dopamine neurons. Consequently, an uninhibited dopaminergic drive to striatal areas underlies the formation of positive symptoms. Multiple factors acting on the excitatory and inhibitory control of cortical neurons in psychosis are likely, but not known in detail. For example, these factors could include neurodevelopmental insults leading to loss of synaptic spines, or the dysregulation of various neurotransmitter and -signaling systems, such as the ECS.

The endocannabinoids anandamide (AEA) and 2-arachidonoylglycerol are synthesized on post-synaptic neuronal membranes in response to activity. They act through the CB1R, one of the most numerous G-protein receptors in the brain, to retrogradely inhibit further neurotransmitter release of mainly glutamatergic and gamma-aminobutyric acid (GABA) synapses.^17^ The ECS has the capacity to modulate the information flow within cortico-striato-thalamic circuits either directly, or through cortical glutamatergic projections to the midbrain. In addition, the ECS contributes to regulation of gonado- and corticotropic endocrine axes, peripheral metabolism, central and peripheral immune functions, as well as functional coupling of neurons and neuroglia. Evidence of increased CSF AEA levels, altered brain CB1R availability, and associations of these measures to the core symptomatology of psychosis, indicates that ECS dysregulation has etiological significance in psychotic disorders.^24^ For example, dopaminergic dysregulation could partly stem from ECS modulation of cortical glutamatergic output.^41–43^ However, the result of no differences in regional CB1R availabilities between female FEP and HC suggests that midbrain dopamine dysregulation driven primarily by cortical ECS dysregulation is not by itself a sufficient etiological model for the emergence of positive symptoms. Notably, the ECS is also poised to modulate information processing downstream to the effects of striatal dopamine dysregulation. The ACC acts as an integrator of emotional and cognitive processing of stress, and its function is known to be modulated by gonadotropic hormones.^44^^,^^45^ Considering that ECS function is regulated by gonadotropic hormone systems, the dysregulation of the ECS can plausibly be thought to be sex specific.^22^^,^^25^

Although the study was underpowered to study the associations of symptoms to V_T_, introducing BPRS_total_ symptom scores as a covariate in to the model of CB1R V_T_ of FEP participants did not alter the level of non-corrected significance of this sex difference. This indicates, that although males and females had different total symptom scores, the ECS differences between males and females are not explained by sampling bias. Also, females and males are known to present with slightly differing degrees of symptom severity, and fully matched symptom severities would not reflect the characteristics of the study population.

A sex related factor influencing the impact of dopaminergic dysregulation may partially account for the sex differences in age of onset and level of functioning. However, it is also plausible to suggest, that the pathophysiological mechanisms leading to dopaminergic dysregulation are multifactorial in nature, and that this etiology seems to be differentially expressed in males and females. Ultimately, confirmatory replication with larger samples of male and female FEP is needed to ascertain whether the result of the direct comparison between FEP participants reflects a true property of the study population. The significance of elucidating the role of the ECS in FEP is highlighted by notions suggesting that pharmacological targeting of this non-dopaminergic signaling pathway could potentially delay the onset of psychosis and/or mitigate its core symptoms, regardless of the level of its etiological contribution.^31^

### Strengths and Limitations

We used state-of-art CB1R imaging methodology with metabolite and injection delay corrected arterial input functions as well as a validated kinetic model to obtain measures of CB1R availability.^46^ The volumes of ROI were smaller in females compared to males, but partial volume effects are not relevant given the size of the ROI compared to the high resolution research tomograph (HRRT) scanner spatial resolution of ~2.7 mm.^47^^,^^48^ Cannabis and other substance use were controlled for as potential sources of bias. Female subjects were required to not use oral hormonal contraceptives, but the phase of individual hormonal cycles was not documented. All of the participants were of Finnish ancestry, and the results may not generalize across races. However, results of the male FEP and HC samples have been partly reported previously,^14^ indicating that male FEP ECS dysfunction was seen in both white and mixed-race samples. Also, we extended the male FEP sample reported previously by including one male FEP subject who previously suffered from cannabis dependence, but who had achieved remission before the PET study. The leave-one-out sensitivity analysis indicates that including or excluding this one subject from the study did not affect the main results. The proportion of non-affective to affective psychotic disorders were similar in the groups of males and females with FEP. However, despite the decent total study sample size and lack of obvious demographic differences between male and female patients, the modest subgroup sizes of this study could have affected our results. A significantly lower mean BPRS total symptom score in females with FEP, compared to males with FEP, could still be argued to be due to sampling bias. Since the availability of CB1R V_T_ has been previously suggested to be inversely associated to positive symptoms, a difference between females with FEP and female HCs might be so small, that it is not detectable here. However, this would not negate the regionally specific effect of ROI*sex*group. Further, the replication design with independent patient and control samples in our previous study reduces the probability of sampling bias as an explanation of these results.^14^ Ultimately, this study was underpowered to fully ascertain or refute a significant difference in a direct comparison between males and females with FEP. Also, a minute difference between females with FEP and HC might also be undetectable with this sample size. It is also important to note, that PET imaging studies on FEP may have limited generalizability due to well-known selection bias.^49^

## Conclusions

Brain cannabinoid receptor type 1 availability is significantly altered in vivo in male patients with FEP, but no significant alterations were found in female patients when compared to matched control subjects. Consequently, we found evidence for a sex-specific and regionally selective change in regional CB1R availability. These results support the view that the neurobiology of neurotransmitter system networks is different in males and females with psychotic disorders. Characterizing this divergence further would deepen our understanding on the etiologies of psychotic disorders, and, importantly, facilitate the development of individualized and perhaps sex-specific treatment strategies.

## Supplementary Material

FMPEP_FEP_SEX_SUPPLEMENT_SchzBull_sbag038
