# Supplementary material for: The Brain Endocannabinoid System is Differentially Regulated in Male and Female Patients with First-Episode Psychosis
Source: Schizophr Bull. 2026 May 5;52(3):sbag038. doi: 10.1093/schbul/sbag038 (PMC13140530; doi:10.1093/schbul/sbag038)
Supplement: FMPEP_FEP_SEX_SUPPLEMENT_SchzBull_sbag038 [file fmpep_fep_sex_supplement_schzbull_sbag038.docx]

SUPPLEMENT

**Symptom and CB1R correlations:**

Neither BPRS_total_ (F(1,15)=0.051, p=0.8) or BPRS_positive_ (F(1,15)=0.409, p=0.53) were significantly associated to V_T_, when introduced as covariates into rmANOVA models including FEP participants (V_T_ ~ ROI*sex + BPRS_total/positive_ + Error(ID/ROI)). Introducing BPRS_total_ as a covariate did not affect the interpretation of the regionally specific difference between male and female FEP (ROI*sex; F(3,45)=3.005, p=0.04; p_FWER_=0.24). Introducing BPRS_positive_ scores as a covariate did not affect the interpretation of the regionally specific difference between male and female FEP (ROI*sex; F(3,45)=2.689, p=0.06; p_FWER_=0.36). The BPRS_positive_ symptom sum score showed inverse associations with ACC V_T_ (R_Pearson_=-0.76, p=0.028), PUT V_T_ (R_Pearson_=-0.76, p=0.028) and THA V_T_ (R_Pearson_=-0.73, p=0.042) in male FEP participants. There were no significant associations to BPRS symptom severity in female FEP participants. However, the associations between BPRS_positive_ and [^18^F]FMPEP-*d_2_* V_T_ in males were not significant after Bonferroni correction for multiple comparisons (ɑ_FWER_=0.4).
